# Analysis of Potential Ischemic Effect of Intravitreal Bevacizumab on Unaffected Retina in Treatment-Naïve Macular Edema Due to Branch Retinal Vein Occlusion: A Prospective, Interventional Case-Series

**DOI:** 10.1371/journal.pone.0162533

**Published:** 2016-09-12

**Authors:** Pukhraj Rishi, Neha Raka, Ekta Rishi

**Affiliations:** Shri Bhagwan Mahavir Vitreoretinal Services, Sankara Nethralaya, Chennai, TamilNadu, India; LV Prasad Eye Institute, INDIA

## Abstract

**Background:**

To study potential ischemic effects of intravitreal Bevacizumab (IVB) on unaffected retina in treatment-naive eyes with macular edema secondary to branch retinal vein occlusion (BRVO) and contralateral eyes secondary to systemic absorption.

**Methods and Findings:**

Prospective, interventional series included 27 treatment-naive eyes with BRVO and macular edema. Exclusion criteria: Eyes with diabetic retinopathy, glaucoma, vasculitides, papilledema or systemic neurologic condition. Subjects underwent complete ophthalmological examination including fluoroscein angiography (FA), optical coherence tomography (OCT) and multifocal electroretinogram (mf-ERG). All subjects received single 1.25 mg/0.05ml IVB injection. Two observers measured all parameters; inter-observer agreements were expressed as kappa values. Paired t-test was used to compare values at baseline and follow-up. The statistical analysis was done using SPSS for Windows, Version 14.0. (Chicago, SPSS Inc.) Presenting mean CFT (central foveal thickness) was 499.5(+/-229.7) μm, mean BCVA (best corrected visual acuity) was 0.64(+/-0.41) logMAR. At last follow-up, mean CFT was 267.9(+/-159.3) μm (P<0.001), 95% CI [127.18, 422.32]; mean BCVA was 0.28(+/-0.24) logMAR. Respectively, mean N1 and P1 amplitudes of mfERG in 'unaffected quadrant' at presentation were -6.10(+/-4.00) nV/deg^2^ and 17.17(+/-11.54)nV/deg^2^; and -5.33(+/-1.30)nV/deg^2^ and 15.29(+/-4.69)nV/deg^2^ at final follow-up (P = 0.631 and 0.197, respectively), (95% CIs [-0.93, 1.42] and [-4.22, 1.08] respectively). In fundus quadrant of fellow eyes corresponding to unaffected quadrant in treated eyes, mean N1 and P1 amplitudes at presentation were -5.39(+/-1.56)nV/deg^2^ and 15.89(+/-3.89)nV/deg^2^; and -5.39(+/-1.90)nV/deg^2^ and 15.9(+/-5.52)nV/deg^2^ (P = 0.380 and 0.208), (95% CIs [-0.57, 1.28] and [-4.1, 1.1]) at last follow-up, respectively. Limitations: This study analysed the effects with a single injection of bevacizumab. However, whether ischemic adverse effects will emerge with repeated IVB injections as a consequence of cumulative dosing needs further investigation. The setting of our study being a tertiary care centre, the numbers of fresh BRVO cases without prior intervention were limited. Thus, the limitations of our study include a small sample size with a small follow-up period. No major ocular/systemic adverse event was observed in the study period.

**Conclusion:**

No evidence of progressive ischaemia attributable to single bevacizumab treatment was observed in this study. However, a larger prospective study involving subjects with cumulative dosing of bevacizumab and a longer follow-up could provide a better understanding of the potential ischaemic effects of bevacizumab or other anti-VEGF agents.

## Introduction

Branch retinal vein obstruction (BRVO) is the second most common retinal vascular disorder after diabetic retinopathy. [1] Visual loss is usually caused by macular edema, macular ischemia, or vitreous hemorrhage. Conventionally, laser photocoagulation has been used to treat macular edema but is now rapidly shifting to intravitreal anti-vascular (VEGF). [2–4] Safety data ranging from histology to functional retinal testing to adverse event reporting in larger trials is continuously accumulating. Various theories and reports suggest variable potential of anti-VEGFs in causing an ischemic effect; most probably due to interference with the action of the natural vasogenic compound i.e. VEGF. [5–8] In mouse retina, a significant increase in apoptosis of cells in the inner and outer nuclear layers, causing reduced thickness of the inner and outer nuclear layers and a decline in retinal function as measured by electroretinograms was noted after 14 days of VEGF neutralisation. [9]

A sudden drop in effective VEGF concentration may be responsible for the closure of the normal capillaries. [10] There are reports of development of anterior ischemic optic neuropathy after intravitreal injections of bevacizumab. [11, 12] Long-term neutralization of retinal VEGF is noted to increase the risk of circulation disturbances in the choriocapillaris. [13] Rouvas et al reported a case of retinal angiomatous proliferation in a patient with AMD treated with a combination of photodynamic therapy (PDT) and intravitreal bevacizumab. [14] Yokomaya et al described a case of extensive occlusion of both retinal arteries and veins 4 weeks after intracameral administration of bevacizumab in a patient with neovascular glaucoma and diabetic retinopathy. [15] Pieramici et al found increased macular ischemia in FFA and increase in the extent of retinal hemorrhages in a patient with perfused CRVO following repeated intravitreal injections of ranibizumab. [16] In order to study the potential ischemic effects of IVB, we undertook a prospective (pilot) study designed to monitor the anatomical and functional status of the normal and affected retinal areas in treatment-naive eyes presenting with BRVO and macular edema. The fellow eye was monitored in all subjects to look for any potential changes due to systemic absorption of IVB.

## Methods

**Fig 1:**
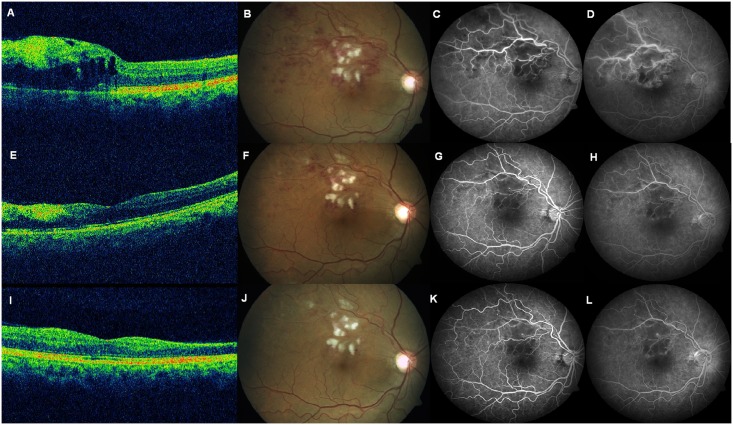
Composite image of OCT, colour fundus photo, and fluorescein angiography in an eye with superotemporal branch vein occlusion. This figure shows findings at baseline (A-D), month 1 (E-H), and month 3 (I-L). At baseline, Optical coherence tomography (OCT) reveals cystoid macular thickening (A) due to superotemporal branch vein occlusion (B), with fluorescein angiography revealing capillary non-perfusion (C) and late leakage (D). At 1 month, OCT reveals resolution of macular edema with inner retinal thinning (E), resolving retinal haemorrhages (F), with reduction in area of capillary non-perfusion (G) and reduced leakage (H). At 3 months, OCT reveals slight increase in macular thickening (I), resolving retinal haemorrhages (J), with further reduction in area of capillary non-perfusion (K) and persistent late leakage (L).

This was a prospective interventional case series. This project was approved by Ethics subcommittee of Vision Research Foundation, Chennai in July 2011. All clinical investigations were conducted according to the principles expressed in the Declaration of Helsinki. Written informed consent was obtained from all the participants. This study included subjects with BRVO and associated macular edema with best corrected visual acuity (BCVA) worse than Snellen’s acuity 6/6 (i.e. logMAR 0.0). Patients were screened for systemic risk factors of BRVO. Each patient underwent complete ophthalmological examination including BCVA, noncontact slit-lamp biomicroscopy, retinal examination with indirect ophthalmoscopy, fundus photography, fundus fluorescein angiography (FFA using Zeiss Digital Angiography software; FF 450plus IR Fundus Camera, VISUPAC Version 4.43), spectral-domain optical coherence tomography (SD-OCT; Topcon 3D OCT-1000) and multifocal electroretinogram (mf-ERG; Veris ScienceTM 5.2.2X, EDI) before receiving intravitreal bevacizumab (IVB) injection. All patients were treated with the same dose of 1.25 mg/0.05ml IVB in affected eye. Following injection, follow-up were scheduled at day 1, month 1, and month 3. Investigations were repeated on each monthly visit (Fig 1). Pre- and post-injection parameters were analyzed to study the effects on structural (OCT and FFA) and functional (BCVA and mf- ERG) outcomes. Analyzed OCT parameters included central foveal thickness (CFT) in affected eye, maximum retinal thickness in affected eye, CFT of contralateral eye, thickness of unaffected part of retina at 2500 microns from fovea in a vertical scan measuring normal retinal thickness (NRT), and signs of acute retinal ischemia like hyper-reflectivity of inner retina and appearance of middle limiting membrane ‘MLM’ sign. [17–19]

**Fig 2:**
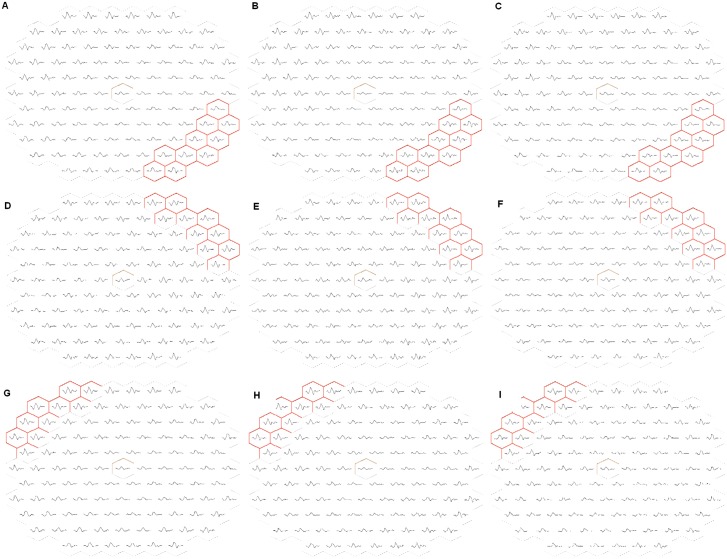
Multifocal ERG plot of a subject with inferotemporal BRVO in left eye. Plot recorded at baseline (A), month 1 (B), and month 3 (C). Corresponding points in the superotemporal quadrant of the affected eye are recorded at same time intervals (D, E, and F, respectively). Corresponding points in the superotemporal quadrant of the contralateral eye are also recorded at same time intervals (G, H, and I, respectively).

Analyzed FFA parameters included outline of foveal avascular zone (FAZ), area of FAZ, outline of half of FAZ in unaffected side of retina (using freehand drawing tool of VISUPAC Version 4.43 software on early fluorescein angiography frames with 50° images), capillary non-perfusion (CNP) areas in unaffected side of retina, CNP areas in the contralateral eye. [20, 21] BCVA was recorded at each visit. Analyzed multifocal electroretinogram (mf-ERG) parameters included N1 amplitude, N1 implicit time, P1 amplitude and P1 implicit time in ‘affected quadrant’ and ‘unaffected quadrant’. [22–26] In the contralateral eye, same parameters were analysed from the quadrant corresponding to the defined ‘unaffected quadrant’ in affected eye. An average of 10 hexagonal points in perifoveal zone (to avoid spill-over as far as possible) was considered as shown in Fig 2. Exclusion criteria ruled out eyes with central retinal vein occlusion (CRVO), diabetic retinopathy, sickle cell retinopathy, systemic vasculitic conditions, papilledema or sequelae of disc edema. The statistical analysis was done using SPSS for Windows, Version 14.0. (Chicago, SPSS Inc.).

## Results

Overall, 27 patients were recruited over a period of 18 months (October 2011 to March 2013). The mean age was 54.4 years (+/-10 years). There were 22 (81.5%) male and 5 (18.5%) female subjects. 18 (66.7%) patients presented with BRVO in right eye, whereas left eye was affected in 9 (33.3%) of the patients. The mean duration of symptoms was 9.7 (+/-9.9) weeks; the most common symptom being diminution of vision—present in 26 (96.30%) patients. Only one patient complained of metamorphopsia and visual field defects. Associated systemic conditions included hypertension (HTN, n = 16), diabetes mellitus (DM, n = 11), hyperlipidemia (n = 5), homocystinuria (n = 1) and ischemic heart disease (IHD, n = 1). Eight (30%) patients had no systemic conditions. None of the patients had previous history of taking any treatment for BRVO i.e. patients were treatment naïve. BCVA at presentation ranged from Snellen’s acuity 6/7.5 to counting fingers (CF). At presentation, 41% eyes were phakic without cataract, 44% had nuclear sclerosis I (NS I), 7% had NS II, and 4% had posterior subcapsular cataract. One eye (4%) was pseudophakic. Mean intraocular pressure (IOP) was 14.1(+/-2.1) mmHg. Optic disc was normal in all eyes. Most common fundus quadrant affected by BRVO was superotemporal quadrant (STQ, 59%); followed by inferotemporal quadrant (ITQ, 41%). Fundus quadrant opposite to BRVO-affected quadrant was studied as the unaffected quadrant. Hence, most common unaffected quadrant studied was ITQ. All subjects were treated with same dose of 1.25 mg/0.05ml IVB. First follow-up (scheduled 1 month post IVB) was at an average of 1.3(+/-0.6) months. Second follow-up (scheduled 3 months post IVB) was at an average of 4.52 (+/-4.4) months. All changes on OCT, FFA, BCVA and mf-ERG parameters during follow-up visits were compared to the baseline using the paired t-test. The demographic details, clinical parameters, and imaging results are summarised in S1 File. Details regarding OCT, FFA, and mf-ERG of study subjects are available at http://dx.doi.org/10.5061/dryad.3cp54.

### OCT Parameters

Inter-observer agreements for measurements of CFT in affected eye at presentation, on 1st follow-up and on 2nd follow-up were kappa value = 0.96, 0.93 and 0.95, respectively. Mean CFT at presentation was 499.5 (+/-229.7) microns. It reduced significantly to 258.3 (+/-150.3) microns on 1st follow-up (P value <0.001), (95% CI [148.41, 333.96]) and 267.9 (+/-159.3) microns (P value <0.001), (95% CI [127.18, 422.32]) on 2nd follow-up (Table 1).

**Table 1:** Measured values for central foveal thickness (CFT) in affected eyes.

Visit	Mean	Standard Deviation	Kappa value	P value for paired t-test in comparison to the values ‘at presentation’	95% Confidence Interval
At presentation	499.5	229.7	0.96	-	
1^st^ follow-up	258.3	150.3	0.93	0.00	(148.41, 333.96)
2^nd^ follow-up	267.9	159.3	0.95	0.00	(127.18, 422.32)

### Maximum Retinal Thickness (MRT) in affected eyes

Inter-observer agreements for the measurements at presentation, on 1st follow-up and 2nd follow-up were kappa value = 0.94, 0.96 and 0.96, respectively. Mean MRT at presentation was 664 (+/-149.5) microns. It reduced significantly to 462.3 (+/-188.7) microns on 1st follow-up (P value <0.001), (95% CI [132.25, 271.23]) and 408.1 (+/-193.6) microns (P value <0.001), (95% CI [164.89, 374.06]) on 2nd follow-up (Table 2).

**Table 2:** Measured values for maximum retinal thickness (MRT) in affected eyes.

Visit	Mean	Standard Deviation	Kappa value	P value for paired t-test in comparison to the values ‘at presentation’	95% Confidence Interval
At presentation	664	149.5	0.94	Not applicable	
1^st^ Follow-up	462.3	188.7	0.96	0.00	(132.25, 271.23)
2^nd^ Follow-up	408.1	193.6	0.96	0.00	(164.89, 374.06)

### Normal Retinal Thickness (NRT) of the affected eyes

Inter-observer agreements for the measurements at presentation, on 1st follow-up and on 2nd Follow-up were kappa value = 0.97, 0.96 and 0.98 respectively. Mean NRT at presentation was 241.9 (+/-18.9) microns. It changed to 241 (+/-19.9) microns on 1st follow-up (P value = 0.49), (95% CI [-1.5, 4.24]) and 235.7 (+/-21.7) microns (P value = 0.34), (95% CI [-3.31, 10.19]) on 2nd follow-up (Table 3).

**Table 3:** Measured values for normal retinal thickness (NRT) in affected eyes.

Visit	Mean	Standard Deviation	Kappa value	P value for paired t-test in comparison to the values ‘at presentation’	95% Confidence Interval
At presentation	241.9	18.9	0.97	-	
1^st^ follow-up	241	19.9	0.96	0.49	(-1.5, 4.24)
2^nd^ follow-up	235.7	21.7	0.98	0.34	(-3.31, 10.19)

### Signs of acute ischemia

None of the OCT scan images showed either development of hyper-reflectivity of inner retina or appearance of ‘Middle Limiting Membrane (MLM)’ sign in either of the follow-up scans.

### CFT of contralateral eyes

Inter-observer agreement for measurements at presentation, on 1st follow-up and on 2nd follow-up were kappa value = 0.97, 0.98 and 0.99, respectively. Mean CFT of contralateral eyes at presentation was 175.6 (+/-18.8) microns. It changed to 177.9 (+/-19.8) microns (P value = 0.06), (95% CI [-5.56, 0.16)]) on 1st follow-up and 187.1 (+/-18.2) microns (P value = 0.28), (95% CI [-8.61, 2.14]) on 2nd follow-up (Table 4).

**Table 4:** Measured values for central foveal thickness (CFT) in contralateral eyes.

Visit	Mean	Standard Deviation	Kappa value	P value for paired t-test in comparison to the values ‘at presentation’	95% Confidence Interval
At presentation	175.2	18.8	0.97	-	
1^st^ follow-up	177.9	19.8	0.98	0.06	(-5.56, 0.16)
On 2^nd^ Follow-up	187.1	18.2	0.99	0.28	(-8.61, 2.14)

## FFA Parameters

### Outline of Foveal Avascular Zone (FAZ)

Inter-observer agreements for the findings at presentation, on 1st follow-up and on 2nd follow-up were kappa value = 0.94, 0.98 and 0.94, respectively. The outline of FAZ as a whole was found to be intact in 6 eyes (22%) and distorted in 21 eyes (78%) at presentation. On 1st follow-up; it remained same in 22 (92%), improved from ‘distorted’ to ‘intact’ FAZ in 2 (8%) whereas none of them worsened from ‘intact’ to ‘distorted’. On 2nd follow-up, it remained same in 100% eyes. There was neither improvement from ‘distorted’ to ‘intact’ nor worsening from ‘intact’ to ‘distorted’ in any of the eyes.

### Area of FAZ

Inter-observer agreements for the measurements at presentation, on 1st follow-up and on 2nd follow-up were kappa value = 0.86, 0.90 and 0.84, respectively. Mean FAZ area at presentation was 0.65 (+/-0.34) mm^2^ and changed to 0.61 (+/-0.21) mm^2^ on 1st follow-up and to 0.57(+/-0.29) mm^2^ on 2nd follow-up. These changes were not statistically significant (P values 0.953 and 0.213, 95% CIs [-0.05, 0.27] and [-0.07, 0.27] respectively). (see Table 5).

**Table 5:** Measured values for area of foveal avascular zone (FAZ) in affected eyes.

Visit	Mean	Standard Deviation	Kappa value	P value for paired t-test in comparison to the values ‘at presentation’	95% Confidence Interval
At presentation	0.65	0.34	0.96	-	
1^st^ follow-up	0.61	0.21	0.93	0.953	(-0.05, 0.27)
2^nd^ follow-up	0.57	0.29	0.95	0.213	(-0.07, 0.27)

### Outline of half of FAZ on the unaffected side of retina (hemi-circumference)

Inter-observer agreements for the findings at presentation, on 1st follow-up and on 2nd follow-up were kappa value = 0.96, 0.96 and 0.94, respectively. Distortion of hemi-circumference of FAZ was classified into three grades. Grade I: no distortion, grade II: distortion <50% of normal outline, and Grade III: distortion >50% of normal outline. At presentation, 22 eyes (82%) were classified into Grade I, 3 eyes (11%) as Grade II, and 2 eyes (7%) as grade III. On 1st follow-up; it remained same in 22 (92%), improved in 2 (8%) and worsened in none. On 2nd follow-up (n = 9); it remained same in 5 (56%), worsened in 4 (44%) and improved in none.

### CNP areas in the unaffected side of retina

Inter-observer agreements for the findings at presentation, on 1st follow-up and on 2nd follow-up was kappa value = 1 at each visit. None of the eyes showed presence of CNP areas on the unaffected side of the retina at presentation. There were no new development of CNP areas post IVB, both on 1st follow-up and 2nd follow-up.

### CNP areas in the contralateral eyes

Inter-observer agreements for the findings at presentation, on 1st follow-up and on 2nd follow-up was kappa value = 1 at each visit. None of the patients showed presence of any leakage or CNP areas in the contralateral eyes at presentation. There were no new development of CNP areas post IVB, both on 1st follow-up and 2nd follow-up.

## BCVA

Mean BCVA at presentation was 0.64 (+/-0.41) logMAR which improved to 0.32 (+/-0.26) logMAR on 1st follow-up and further improved to 0.28 (+/-0.24) logMAR on 2nd follow-up. Individually; BCVA on 1st follow-up improved in 21 (78%), remained same in 3 (11%) and worsened in 3 (11%) eyes. On 2nd Follow-up, compared to the BCVA at presentation; BCVA improved in 12 (81%), remained same in 3 (12%) and worsened in 1 (6%) eyes.

## mf-ERG Parameters

### ‘Affected’ quadrant

Measured values of mean N1 and P1 amplitudes in affected eyes at presentation, on 1st follow-up and 2nd follow-up are listed in Table 6. Interval changes were not significant. The mean N1 and P1 implicit times at presentation were 18.64 (+/-2.21) ms and 33.95 (+/-2.91) ms, respectively. On 1st follow-up, the mean values changed to 17.28 (+/-2.20) ms and 33.65 (+/-3.44) ms, respectively (P values = 0.016 and 0.438, 95% CIs [3.27, 9.69] and [-0.79, 1.75]). The reduction in N1 implicit time was statistically significant but changes in P1 implicit time were not statistically significant. On 2nd follow-up, the mean values changed to 18.23 (+/-2.78) ms and 33.7 (+/-4.01) ms, respectively (P values = 0.328 and 0.147, 95% CIs [-1.52, 3.86] and [-1, 5.26]). These changes were not statistically significant (Table 7).

**Table 6:** Measured values of N1 and P1 amplitudes in the 'affected' quadrant.

Visit	N1 Amplitude			P1 Amplitude		
	Mean (nV/deg^2^)	Standard Deviation	P value for paired t-test in comparison to values ‘at presentation’ (95% Confidence Interval)	Mean (nV/deg^2^)	Standard Deviation	P value for paired t-test in comparison to values ‘at presentation’ (95% Confidence Interval)
At presentation	-3.88	1.25	-	10.14	3.09	-
1^st^ follow-up	-3.85	1.09	0.69 (-2.11, -0.23)	10.45	3.38	0.20 (-2.01, 0.45)
2^nd^ follow-up	-3.8	0.99	0.19 (-0.48, 1.94)	10.2	3.14	0.11 (-5.11, 0.71)

**Table 7:** Measured values of N1 and P1 implicit times in the 'affected' quadrant.

Visit	N1 Implicit Time			P1 Implicit Time		
	Mean (milli-seconds)	Standard Deviation	P value for paired t-test in comparison to the values ‘at presentation’	Mean (milli-seconds)	Standard Deviation	P value for paired t-test in comparison to the values ‘at presentation’
At presentation	18.64	2.21	-	33.95	2.91	-
1^st^ follow-up	17.28	2.20	0.016 (3.27, 9.69)	33.65	3.44	0.438 (-0.79, 1.75)
2^nd^ follow-up	18.23	2.78	0.328 (-1.52, 3.86)	33.7	4.01	0.147 (-1, 5.26)

### ‘Unaffected’ quadrant

Measured values of mean N1 and P1 amplitudes in unaffected quadrant of affected eyes at presentation, on 1st follow-up and 2nd follow-up are listed in Table 8. Interval changes were not significant. Measured values of mean N1 and P1 implicit times in unaffected quadrant of affected eyes at presentation, on 1st follow-up and 2nd follow-up are listed in Table 9. Interval changes were not significant.

**Table 8:** Measured values of N1 and P1 amplitudes in the 'unaffected' quadrant.

Visit	N1 Amplitude			P1 Amplitude		
	Mean (nV/deg^2^)	Standard Deviation	P value for paired t-test in comparison to the values ‘at presentation’	Mean (nV/deg^2^)	Standard Deviation	P value for paired t-test in comparison to the values ‘at presentation’
At presentation	-6.10	4.00	-	17.17	11.54	-
1^st^ follow-up	-5.06	1.80	0.337 (-4.46, -0.67)	14.15	4.22	0.369 (-2.33, 5.97)
2^nd^ follow-up	-5.33	1.30	0.631 (-0.93, 1.42)	15.29	4.69	0.197 (-4.22, 1.08)

**Table 9:** Measured values of N1 and P1 implicit times in the 'unaffected' quadrant of affected eyes.

Visit	N1 Implicit Time			P1 Implicit Time		
	Mean (milli-seconds)	Standard Deviation	P value for paired t-test in comparison to the values ‘at presentation’	Mean (milli-seconds)	Standard Deviation	P value for paired t-test in comparison to the values ‘at presentation’
At presentation	16.61	1.39	-	30.80	2.03	-
1^st^ follow-up	16.24	2.08	0.321 (2.22, 8.15)	30.31	2.31	0.090 (-0.16, 2.02)
2^nd^ follow-up	16.06	1.05	0.213 (-0.66, 1.83)	29.29	1.49	0.050 (0.02, 4.53)

### Contralateral eyes

Measured values of mean N1 and P1 amplitudes in corresponding quadrant of unaffected eyes at presentation, on 1st follow-up and 2nd follow-up are listed in Table 10. Interval changes were not significant. Measured values of mean N1 and P1 implicit times in corresponding quadrant of unaffected eyes at presentation, on 1st follow-up and 2nd follow-up are listed in Table 11. Interval changes were not significant.

**Table 10:** Measured values of N1 and P1 amplitudes in the corresponding quadrant of 'unaffected' contralateral eyes.

Visit	N1 Amplitude			P1 Amplitude		
	Mean (nV/deg^2^)	Standard Deviation	P value for paired t-test in comparison to the values ‘at presentation’	Mean (nV/deg^2^)	Standard Deviation	P value for paired t-test in comparison to the values ‘at presentation’
At presentation	-5.39	1.56	-	15.89	3.89	-
1^st^ follow-up	-5.14	1.71	0.793 (-3.06, -0.47)	15.29	4.15	0.782 (-1.51, 1.16)
2^nd^ follow-up	-5.39	1.90	0.380 (-0.57, 1.28)	15.9	5.52	0.208 (-4.1, 1.1)

**Table 11:** Measured values of N1 and P1 implicit times in the corresponding quadrant in contralateral eyes.

Visit	N1 Implicit Time			P1 Implicit Time		
	Mean (milli-seconds)	Standard Deviation	P value for paired t-test in comparison to the values ‘at presentation’	Mean (milli-seconds)	Standard Deviation	P value for paired t-test in comparison to the values ‘at presentation’
At presentation	16.16	1.25	-	29.47	1.56	-
1^st^ follow-up	16.31	1.61	0.800 (-1.78, 7.59)	29.78	1.88	0.902 (-0.84, 0.75)
2^nd^ follow-up	15.96	1.02	0.114 (-0.23, 1.63)	29.4	1.51	0.797 (-0.93, 1.16)

## Discussion

Of late, there have been several animal studies, case reports and theories that suggest possible ischemic effect of anti-VEGF agents on ocular structures. Considering the vast usage of anti-VEGF agents in today’s ophthalmic practices it becomes imperative to identify the potential ischemic effects of these therapeutic agents on normal retina, if any. There is little description about fluorescein angiographic patterns in the BRAVO and CRUISE studies although vision and OCT results were very encouraging. [27, 28] The rationale of this prospective study was to gather evidence of any potential ischemic effects of IVB on the retina using anatomic and functional investigations like OCT, FFA and mf-ERG. The vitreous half-life of IVB being 6.7 days, the elimination period of the drug ranges from 33.5 to 46.9 days (i.e. 5 to 7 half-lives). Hence, we followed up patients at 1 month and 3 months after administration of single dose of IVB which ensured complete elimination of drug from the eye. This duration of follow-up appears to be adequate to study the potential adverse effects associated with one-time administration of IVB. In a recent study, Campbell et al analysed the systemic adverse events with intravitreal injection of vascular endothelial growth factor inhibitors in a nested case-control study. They found that intravitreal injections of bevacizumab and ranibizumab were not associated with significant risks of ischaemic stroke, acute myocardial infarction, congestive heart failure, or venous thromboembolism. [29]

In our study, improvement in BCVA corresponded to significant reduction in CFT and macular edema as evident by OCT measurements. There was no significant change in thickness of unaffected retina following IVB. Thus, OCT changes were not suggestive of any structural changes caused due to any potential ischemic effects of IVB. Changes in FFA did show an improvement in distortion of the outline of FAZ after receiving IVB. The hemi-circumference of FAZ in the unaffected retinal side also did not show adverse changes in any of the eyes at 1 month and 56% eyes at 3 months after IVB. This worsening of the hemi-circumference of FAZ outline 3 months after IVB could be partially attributed to chronicity of macular edema. There was no new development of any CNP areas either after 1 month or 3 months of IVB that could provide any evidence of retinal ischemia. mf-ERG findings in affected quadrant showed significant reduction in N1 implicit time suggestive of an improved functional status of affected quadrant of retina 3 months after IVB. N1, P1 amplitudes and P1 implicit time showed no significant changes at either 1 month after IVB or 3 months after IVB in the affected quadrant of retina. In the unaffected part of retina, none of the readings i.e. N1, P1 amplitudes or N1, P1 implicit time showed any significant change either after 1 month or after 3 months of IVB. Thus mf-ERG did not provide any evidence of deterioration of functional status of retina due to any potential ischemic effect of IVB. The measurements, findings and interpretations drawn from the investigations of the contralateral eye also did not show any evidence of potential ischemic effect in the contralateral eye due to systemic absorption of IVB.

Hence, no evidence of progressive ischaemia attributable to bevacizumab treatment was observed in this series of patients. However, whether ischemic adverse effects will emerge with repeated IVB injections as a consequence of cumulative dosing will need further investigation. The setting of our study being a tertiary care centre, the numbers of fresh BRVO cases without prior intervention were limited. Thus, the limitations of our study include a small sample size with a small follow-up period. A larger prospective study with a longer follow-up period would provide better statistical significance to our results.

## Supporting Information

S1 File Flowsheet. Flowsheet listing the demographic, clinical and investigational details of all study subjects.
(XLSX)
